# The Influence of Fixed Dental Prostheses on the Expression of Inflammatory Markers and Periodontal Status—Narrative Review

**DOI:** 10.3390/medicina59050941

**Published:** 2023-05-13

**Authors:** Lucian Paul Dragomir, Flavia-Mirela Nicolae, Dorin Nicolae Gheorghe, Dora Maria Popescu, Iuliana Manuela Dragomir, Lidia Boldeanu, Virgil Mihail Boldeanu, Mihai Raul Popescu

**Affiliations:** 1 Department of Occlusology and Fixed Prosthetics, Faculty of Dental Medicine, University of Medicine and Pharmacy of Craiova, 200349 Craiova, Romania; 2 Research Center of Periodontal-Systemic Implications, Department of Periodontology, Faculty of Dental Medicine, University of Medicine and Pharmacy of Craiova, 200349 Craiova, Romania; 3 PhD Student, Doctoral School, University of Medicine and Pharmacy of Craiova, 200349 Craiova, Romania; 4 Department of Public Health and Management, Faculty of Medicine, University of Medicine and Pharmacy of Craiova, 200349 Craiova, Romania; 5 Department of Microbiology, Faculty of Medicine, University of Medicine and Pharmacy of Craiova, 200349 Craiova, Romania; 6 Department of Immunology, Faculty of Medicine, University of Medicine and Pharmacy of Craiova, 200349 Craiova, Romania

**Keywords:** periodontitis, dental prosthesis, inflammation, inflammation mediators

## Abstract

A dental prosthesis will only be successful if the restoration lasts for a long period and does not cause any illness. The presence of permanent prosthetic restorations has been linked to an increased risk of periodontal infections, according to a large body of research that has been gathered. When chronic inflammation is brought on by fixed prosthetic constructions, both cellular and noncellular immunity are activated as adaptive immune mechanisms. It has previously been stated that both clinically adequate and inadequate restorations might cause gingival inflammation. Areas surrounding the abutment teeth presented periodontal pockets, attachment loss, congestion, bleeding on probing, and gingival hyperplasia after fixed restorations were removed. The depth of pockets, bleeding on probing, and bone loss are all closely correlated with disease’s severity and IL-1β concentration in gingival crevicular fluid; IL-1β shows higher values in disease sites than in healthy ones. hs-CRP and TNF-α blood levels showed a considerable reduction one day after fixed restorations were applied, in comparison with the pre-treatment values. Collaboration between prosthodontists and periodontists is essential for a good treatment outcome since it will increase the restoration’s lifespan, enhance periodontal health, and improve the quality of life for dental patients.

## 1. Introduction

Clinical studies have shown that fixed dental prostheses are essential for tooth restoration or tooth replacement. The patients’ quality of life and oral health are improved through prosthetic rehabilitation utilizing fixed dental prosthesis [1]. The primary objective of restorative dental therapy is to restore the aesthetic, masticatory, and periodontal function of missing teeth. Although periodontal health is influenced by the restoration, it is crucial for a successful prosthesis [2]. Comparative evaluation of prosthetic structures is a difficult and incompletely understood topic [3]. One study with a follow-up of 50 years found that only 3.9% of 223 fixed restorations failed, and these failures were caused by periapical periodontitis rather than periodontal issues. They showed that individuals with good oral hygiene who were followed up annually for this period of time had a high survival percentage for indirect restorations. This implies the value of ongoing maintenance, follow-up, and oral hygiene in clinical practice. However, the study was biased in the direction of individuals who had excellent oral hygiene habits and attended regularly [4].

A dental prosthesis will only be successful if the restoration lasts for a long period and does not cause any illness. The presence of permanent prosthetic restorations has been linked to an increased risk of periodontal infections, according to a large body of research that has been gathered [5,6]. Prosthetic dentistry and periodontology are closely related, as prosthodontic restorations’ durability is significantly influenced by periodontal health; on the other side, faulty prosthesis could promote the progression of periodontal disease [3,7]. Periodontal tissues should be examined for their state of oral cleanliness, gingival health, and periodontal health prior to beginning prosthetic therapy. The discrepancy of margin causes increased dental biofilm buildup, microleakage, hypersensitivity, margin discoloration, increased gingival crevicular fluid flow, recurrent caries, pulp infection, and, finally, periodontal lesions and bone loss, which can result in the failure of prosthetic treatment or even tooth loss [3,5,8].

Periodontitis is a chronic inflammatory disease that affects the tooth-supporting tissues, gingiva, cementum, periodontal ligaments and alveolar bone. It is brought on by an imbalance in the oral microbiota mediated by the hosts’ immuno-inflammatory abnormal response, and there are numerous studies suggesting a close interdependence between periodontal disease and systemic health or treatment for systemic diseases, or between the orthodontic treatment and the periodontium, through the influence of the inflammatory response [9,10,11,12]. Finally, loss of teeth occurs as a result, necessitating prosthetic care [2,7]. The stomatognathic system is biomechanically impaired by a delay in prosthetic therapy, and this has negative effects on the patients’ general health and behavior [3,7]. 

When chronic inflammation is brought on by fixed prosthetic constructions, both cellular and noncellular immunity are activated as adaptive immune mechanisms. These immune systems play a crucial part in the healing process with the regrowth and reconstruction of damaged tissues as well as in the subsequent limiting of the inflammatory response. To restore the injured tissue to equilibrium, self- and acquired immune processes should be coordinated [13]. It is therefore possible to speculate that intrinsic (genetic) or extrinsic (microbiological) variables may be to blame for altering the host’s reaction and predisposing patients to the development of periodontal diseases at sites restored with dental crowns [6]?

### Aim

With the help of literature research, our main focus is to highlight various inflammatory markers from gingival crevicular fluid, peri-implant crevicular fluid, saliva and blood, and their variation in the presence of fixed prosthetic works. The secondary purpose is to emphasize the periodontal status in these patients in order to create a state of the art on the subject.

## 2. Materials and Methods

### 2.1. Search Strategy

The two complementary search strategies used for this narrative review: (i) pathogenic interactions between fixed dental prostheses and periodontal disease, including clinical aspects; and (ii) inflammatory markers in gingival and peri-implant crevicular fluid, saliva, and blood in patients wearing fixed dental prostheses. The databases used for the search were PubMed/Medline, Scopus, Google Scholar, and Science Direct for the period. During the search, the following terms were used in different combinations: “periodontal disease”, “periodontitis”, “inflammation”, “dental prostheses”, “prosthetic crown”, “dental restoration”, “dental abutment”, “fixed dental restoration”, “fixed partial dentures”, “dental crown”, “periimplantitis”, “saliva”, “fixed dental prosthesis”, “blood”, “serum”, “implant prostheses”, “gingival crevicular fluid”, “dental bridge”, “dental crown”. 

### 2.2. Inclusion and Exclusion Criteria for the Selected Studies, Data Extraction, Information Structuring and Review Writing

The included articles had to fulfill the following requirements: in extenso publication, full-text accessibility, and English language writing. Self-reported studies, abstracts, letters and editorials were all excluded from the studies that were generated by the research. Duplicate studies were eliminated after additional evaluation of the papers chosen for inclusion.

Peer-reviewed, full-length English language articles published between 1982 and 2022 made up the selected studies (articles and reviews). The selected publications’ abstracts were initially reviewed for potential inclusion in the research, and then the complete texts of those articles were examined. The papers that were not focused on the proper topic were eliminated. 

Information was organized into sections once relevant research was collected and thoroughly examined for this review. The data was structured in order to offer a comprehensive understanding of the bi-directional pathogenic connections between periodontitis and fixed dental prosthesis, emphasizing the importance of various inflammatory markers.

## 3. Results and Discussion

### 3.1. Clinical Aspects

The anatomy, placement, and relationships of teeth within dental arches are a few of the variables that have been linked to plaque retention, gingivitis, and periodontitis. It has been suggested that the presence, design, manufacture, delivery, and materials of tooth-supported prostheses may have an impact on the periodontium (Table 1). Typically, these factors are connected to localized increases in plaque accumulation and, less frequently, to traumatic and allergic reactions to dental materials. This topic was the subject of a workshop, serving as a support for the new classification of periodontal disease, thus emphasizing the importance of fixed dental prosthesis on periodontal tissues [5].

It has been hypothesized since 1982 that bacterial plaque is necessary for the development of inflammation of the mucosa next to the bridge pontics. Researchers examined fixed dental bridges both before and after professional hygiene methods and discovered that the clinical symptoms of inflammation subsided after using curettes, fine finishing strips, rubber cups, interdental brushes, and dental floss at various time intervals [14]. Conversely, it was shown that regardless of the type of resin used to make the prosthesis, placing temporary crowns leads to greater accumulation of plaque [5]. A previous study also highlighted the importance of dental hygiene. It concluded that tooth-supported and implant-supported crowns with intracrevicular margins were not susceptible to negative gingival and microbiological reactions in patients with good dental hygiene. Six months after the dental prostheses were luted in place, all crown/abutment combinations tested showed favorable soft tissue responses as indicated by gingival redness, swelling, and bleeding [15]. 

Periodontium destruction may occur from prosthodontic operations required for producing fixed prostheses. Gingivitis, gingival recession, and periodontitis can all be generated by techniques and/or materials such as crown preparation, impressions, gingival displacement during impressions, temporary prosthesis, and luting agents [5].

In Passariello et al.’s study, as patients with gingivitis and periodontitis had more prosthetic crowns (1.55 and 1.68 per patient, respectively) than patients with healthy periodontal tissues (1.06 per patient), the authors concluded that possessing dental crowns may have a negative impact on periodontal conditions. The number of metal-ceramic crowns per participant was positively correlated with the presence of localized periodontal pathology [6]. These findings are in accordance with those of The Korea National Health and Nutrition Examination Survey (KNANES VII) from 2016–2018: participants with 6–10 and 11 prosthetic crowns had 1.24 and 1.28 times higher prevalence of periodontitis, respectively, than patients with no prosthetic crowns. The results of this study show that the number of prosthetic crowns present in adults is related to the prevalence of periodontitis [16]. 

It was reported that a single crown application did not statistically alter the plaque index or gingival index after 14 days, respectively, six months following its application. During the period, however, the same parameters showed significant modifications when the patients received fixed partial dentures [17]. In a different study, the patients had their dental crowns examined three to six years after they had been placed. When compared to healthy sites, experimental sites with periodontal disease had higher mean visible plaque index values, according to the examination of clinical data. In the control sites, there were no differences between the groups [6].

In order to reduce the likelihood of gingival recession, the anatomy of the periodontium of teeth receiving crowns should be examined. This is because the presence of an initial shallow periodontal pocket and narrow band of gingiva had a negative impact on the level of periodontal attachment following crown delivery [5]. Although research has shown that gingival inflammation is more common in the areas around crowns, there has not been any evidence of an increase in inflammation compared to periodontal health prior to the placement of crowns or fixed partial dentures [18]. In Passariello’s study, patients underwent examinations 3–6 years after dental crowns were placed. Periodontally-affected sites displayed higher mean gingival bleeding index values than healthy ones, and statistical analysis showed that the differences were both obvious and significant at experimental and control sites [6]. It has previously been stated that both clinically adequate and inadequate restorations might cause gingival inflammation. Reports indicating deeper mean probing depths for crowned teeth, which often tended to be less than 1 mm higher than control teeth, should, however, be questioned due to the limitations in the precision and reproducibility of these measures. After the placement of a prosthetic crown, horizontal bone loss was not increased. The rate of attachment loss or surrounding bone loss was also not accelerated by crowns or fixed partial dentures [18].

A direct restoration with subgingival margins can be linked to localized gingivitis and deeper probing, according to research that is currently available. Particularly for bigger overhangs, a direct or indirect restoration with overhanging borders may be linked to interproximal bone loss, localized gingivitis, and an increase in pocket depth [5].

One study found no negative effects on gingival health following the placement of properly fitted dental crowns for the restoration of permanent molars in children with adequate oral hygiene. Their implantation reduced bleeding on probing, the depth of probing pockets, the inflammation, and increased gingival color. Thus, it was proposed to use these crowns to restore young permanent first molars with severe caries [19].

Diabetes patients experience oral issues more frequently than their non-diabetic counterparts, which might occasionally necessitate fixed-dental prostheses. The most frequent negative effects of dental prostheses are gingival inflammation and various types of periodontal disease. Gingival index, periodontal pocket depths and bleeding on probing in the diabetic group statistically increased at 3 months, respectively, 6 months post-application of prostheses. The outcomes of full veneer crowns in diabetes individuals were shown to be less favorable than those in non-diabetic patients in terms of periodontal health [20].

Cross-sectional studies have shown that an indirect restoration with subgingival margins could be related to gingivitis, especially when self-performed plaque management and periodontal care methods are left out. Subgingival prosthesis margins, however, do not seem to serve as plaque-retentive factors that contribute to gingivitis in longitudinal studies when patient compliance is attained and self-performed plaque management and periodontal maintenance procedures are detailed. When subgingival margins are used in the prosthetic design, it appears that patient plaque management and periodontal care compliance are of utmost significance to preserving the health of the periodontium [5].

### 3.2. Inflammation in Periodontal Tissues

According to another study, areas surrounding the abutment teeth presented periodontal pockets, attachment loss, congestion, bleeding on probing, and gingival hyperplasia after fixed restorations were removed. A hyperplasic reaction occurred on the epithelium’s surface, and at the level of the chorion, an inflammatory chronic reaction was evident, with CD3+ T-lymphocytes making the majority of the cell population [21].

Clinical signs of inflammation, including pain, bleeding of varying degree, gum sensitivity, and halitosis, are present in patients with inflamed periodontal tissues [3,22]. If the hygienic state is not conserved by plaque removal, a permanent prosthetic restoration may worsen the periodontal status of the mucosa under the pontic. In order for the dental structure to survive, patients’ compliance with the maintenance of excellent hygienic practices is crucial [3,23,24]. 

Local endotheliocytes and leukocytes respond to the dental plaque at the borders of the prosthetic restoration, which causes the early development of the lesion. These microorganisms’ metabolic byproducts stimulate junctional epitheliocytes, stimulating the production of cytokines and neuropeptides, which causes blood vessels to expand [7,25,26]. Neutrophils, macrophages, plasma cells, lymphocytes, and mast cells are among the many different cells that move to the diseased foci as the pathological process progresses. The self to the acquired immune response switches over when the pathogenic foci are developed. IgG3 and IgG1 subtypes of B lymphocytes are also present, and plasma cells, macrophages, but also B and T lymphocytes are the predominant lymphocyte types [3,27]. Both blood flow disturbance and an increase in collagenolytic activity are seen. Furthermore, fibroblasts produce additional collagen at a higher rate [3,7,13]. 

It is defined as moderate to severe gingivitis at this clinical phase as it is accompanied by gingival bleeding, abnormalities in shape and gingival color. Clinically, periodontitis develops as a result of these lesions progressing. Both clinically and histologically, irreversible periodontal attachment and alveolar bone loss are found at this stage. Periodontal pockets emerge as the inflammation progresses [13,25,28]. If preventative actions are not taken, inflammatory processes in the periodontal tissues might lead to further pathological changes and premature tooth loss [3,27,29]. 

In addition, individuals with periodontal disease have a significant number of polymorphonuclear leukocyte cells (PMNs) in the gingival pockets. According to various studies, leukocytes made up 47% of the gingival crevice’s cells, with PMNs making up 98% of those cells. In particular, as the inflammation progresses, the total number of cells rises with a difference in PMN (95–97%), mononucleocyte (2–3%), and lymphocyte (1–2%) quantities [3,13].

Dental alloys have the potential to release metal ions and metal particles, which can be detected locally in plaque or periodontal tissues, as well as in other distant organs and tissues. The effects of some of these ions (nickel, palladium, copper, and titanium) on cell count, viability, function, and the release of inflammatory mediators have been demonstrated through in vitro research, although it is uncertain how these ions may affect gingivitis and periodontitis [5].

One of the major metal components in orthodontic and prosthetic devices is nickel (Ni). IL-1β was used to create a pro-inflammatory milieu, and varied Ni doses were used to activate the human gingival fibroblasts. Both Ni concentrations (including clinical average Ni levels) in conjunction with an IL-1β-induced inflammation led to a considerable rise in pro-inflammatory markers and matrix metalloproteinases (MMPs), showing a synergistic impact enhancing inflammation and tissue degradation [30]. The anatomy, placement, and relationships of teeth within dental arches are a few of the variables that have been linked to plaque retention, gingivitis, and periodontitis. It has been suggested that the presence, design, manufacture, delivery, and materials of tooth-supported prostheses may have an impact on the periodontium. Typically, these factors are connected to localized increases in plaque accumulation and, less frequently, to traumatic and allergic reactions to dental materials [5].

### 3.3. Inflammatory Markers in Gingival Crevicular Fluid 

In a healthy gingival crevice, gingival crevicular fluid (GCF) is a physiologic transudate that is present in a small amount. With periodontal inflammation, this amount rises and forms an inflammatory exudate. It is a potential diagnostic and prognostic fluid that offers numerous benefits, including site-specificity, noninvasiveness, and the presence of numerous microbial and host mediators, including pro-inflammatory cytokines like IL-1β, IL-6, IL-8, TNF-α and MMPs [2,31,32,33]. The concentration of inflammatory mediators in GCF suggests the presence of host factors that lead to the development of periodontal alterations at sites restored with dental crowns [6] (Table 2).

Heboyan et al. discovered that the initial state of the periodontium had an impact on the amount of GCF before the hard tissues of the tooth were prepared. The volume of gingival fluid was substantially lower when there was evidence of a mild lesion to the periodontal tissues, but the presence of the highest volume of gingival fluid was seen when there were signs of a severe lesion. During restoration, the GCF volume increases within the first six months of the artificial crown’s fixation, which supports the amplification of periodontal inflammatory processes. After that, over the course of up to 12 months, the GCF secretion progressively returns to a normal index that is similar to the data acquired before the restoration [34]. Moreover, poor oral hygiene may have contributed to certain patients’ increased GCF a year following the fixation of a metal-ceramic prosthesis, which influenced statistics for all groups. One year after the prosthodontic structures were fixed, around 30% of the patients presented poor oral hygiene [34]. Conversely, an older study found that after the quantitative examination of GCF, the degree of gingival inflammation was similar in the tissue around restored and unrestored contralateral teeth [35].

In comparison to data collected prior to restoration, an elevation in GCF pH is shown both in the first six months and a year later after the restoration of masticatory function by artificial crowns. According to these results, the improvement of metabolic processes in the oral cavity causes the pH of the oral fluid to normalize as a result of treatment and preventative measures [34]. 

The application of dental crowns is a risk factor for the development of inflammation, limited to the gingiva or diffused to the periodontium and causing localized damage to periodontal tissues in a group of periodontally-healthy subjects with comparable conditions of oral hygiene at natural teeth and by a comparable general periodontal status [6]. Most experts agree that the risk is mostly caused by the crown’s interference with proper hygiene, which leads to an excessive plaque accumulation that encourages the colonization of harmful bacteria and supports inflammation. The higher visible plaque index scores obtained at both experimental and control sites of patients with periodontal pathology could be an outcome rather than a cause of the presence of higher levels of IL-1β and other cytokines because it is known that the presence of an altered inflammatory milieu promotes the growth of some bacterial species’ bacterial biofilms [6].

Since it stimulates blood flow, attracts neutrophils, promotes other collagenolytic enzymes that destroy tissues and bones, and amplifies other inflammatory mediators such prostaglandin PGE2 and IL-6, IL-1β plays a crucial role in periodontitis’ pathogenesis. The depth of pockets, bleeding on probing, and bone loss are all closely correlated with disease’s severity and IL-1β concentration in GCF; IL-1β showed higher values in disease sites than in healthy ones [2,43,44]. According to Abo-Elmagd et al., the levels of IL-1β were significantly lower at 2 weeks and 4 weeks, respectively, following the application of a temporary or permanent fixed prosthesis in comparison with the values recorded in the first 3 days after the tooth was prepared [2]. Another study showed that 90 days after the cementation of a single crown, the levels of IL-1β were significantly reduced in comparison with day 0 or day 45, depending on the material of the crown [36]. 

Another study examined the levels of IL-1β in a group of children aged 4 to 8 and found that, compared to natural teeth, the levels were lower in cases where the teeth received zirconia dental crowns. Compared to healthy teeth, stainless steel crowns showed greater levels of IL-1β [37].

In one study [38], teeth that had dental crowns placed 3–6 years earlier were examined. Although it is clear that there are significant differences in the levels of IL-1β, IL-6, and TNF-α between healthy sites and periodontally-affected sites, the presence of a significant difference in the level of IL-1β between the control sites of periodontally-healthy sites and periodontally-affected sites was unexpected and suggests an altered reactivity of unknown origin in the absence of clinical evidence of inflammation at these sites [6]. When fixed crown restorations and control teeth were compared, differences in GCF IL-1α and IL-8 concentrations were found but were statistically not significant [38]. 

The inflammation can be reduced in terms of home care by utilizing powered irrigation on a regular basis, either with or without an antibacterial agent. Interleukin-1β and prostaglandin E2, which are linked to degenerative changes in inflamed tissues and bone resorption, were significantly reduced [8,45].

One of the most investigated and efficient possibilities for differentiating between periodontal health and disease is matrix metalloproteinase-8 (MMP-8) collagenase-2/neutrophil collagenase. The biomarker aMMP-8 has been proven to be effective and reliable in detecting the breakdown of periodontal tissue. Moreover, it was decided that the aMMP-8 biomarker was more successful in identifying subclinical periodontitis in teenagers than traditional bleeding on probing [46]. In a group of patients with healthy periodontal tissues, one study examined the levels of IL-1β, interleukin-1 receptor antagonist (IL-1ra), and matrix metalloproteinase aMMP-8 in GCF from teeth restored with fixed prostheses and compared the findings with those found in control teeth without restorations. It was unable to distinguish between an unrestored control tooth and a tooth that had received a dental crown in terms of the inflammatory response. The authors concluded that the materials utilized in the prosthesis, when combined with established clinical techniques, do not cause a detectable inflammatory reaction in the gingiva during a 10-day gingivitis experiment [5,39].

Immune and epithelial cells both release resistin, a hormone peptide generated from adipose tissue. Resistin can act as a pro-inflammatory mediator and is a key marker for inflammatory disorders, according to recent research [47]. Human cartilage glycoprotein-39 (YKL-40) is a recently identified inflammatory factor with several tissue origins. It can be produced endogenously by inflammatory cells including neutrophils and macrophages, as well as by chondrocytes in the joints and synovial cells. Mature neutrophils store it, and it is then released via exocytosis in response to inflammatory stimuli [48]. Moreover, aspartate aminotransferase (AST) and alkaline phosphatase (ALP), which are functional enzymes of osteoblasts, have been linked to periodontal inflammation and may serve as early indicators of periodontitis [40,49]. Zhang reported in a recent study that YKL-40, resistin, AST, and ALP levels significantly increased one year after the fixed restoration of mandibular premolars compared to their pre-restoration values [40].

According to Shang et al., conventional fixed dental prostheses substantially raised the amount of GCF TNF-α, IL-6, sulcus bleeding index, and probing depth [41]. As compared to healthy areas and contralateral controls, the presence of periodontal disorders was also linked to a considerable increase in the secretion of the inflammatory cytokines, IL-1β, IL-6, and TNF-α [6]. One study aimed to compare and contrast the levels of TNF-α in GCF over the course of a 28-day period following the use of various techniques to achieve gingival retraction during dental prostheses impression. There was a rise of almost 35% between the patient’s initial assessment and the results obtained 30 min after applying the retraction cord (13.24 pg/mL). A 95% rise was seen after 7 days, and a 64.2% decline was observed at 28 days. It seems notable that at the end of the fourth trial week, the TNF-α level was still significantly increased, probably due to gingival retraction. Attachment loss may result if cytokine levels remain high over an extended length of time. After retraction and delivery of the final prosthesis, attachment loss or gingival recession might impair the periodontal health of the involved tooth in addition to the aesthetics of the restoration [42].

### 3.4. Inflammatory Markers in Peri-Implant Crevicular Fluid

There is evidence suggesting that the design of the prosthesis (over-contoured restorations, occlusal overloading, opening of inter-proximal contacts) is a major risk factor for developing peri-implantitis [50] (Table 3). Levels of biomarkers linked to tissue damage and inflammation were measured in peri-implant crevicular fluid (PICF) during initial implant healing, one week after delivery of the crown, at three months, and at six months. They found that throughout the observation period, MMP-13 levels rose whereas CRP, TGF-β, IL-1β, IL-6, IL-8, MIP-1a, osteopontin, and osteoactivin protein expression significantly declined [51].

Yaghobee et al. found a significant difference between the level of Il-1β in GCF of healthy teeth and PICF. IL-1β is present at a higher concentration in PICF than GCF, which could be due to the application of fixed prostheses. In both GCF and PICF, it appears that there is a positive correlation between the IL-1β level and clinical parameters, such as plaque index, gingival index, pocket depth, and bone loss [52].

Nitrites and myeloperoxidase (MPO) are a couple of the molecules and enzymes that are thought to change during inflammation. Yet, it is still unclear the precise function they play in the inflammatory reactions around dental implants and natural teeth. As a result, one study [53] compared the concentrations of MPO and nitrites in the proximity of dental implants who underwent prosthetic rehabilitation and healthy teeth. In cases of periodontitis and gingivitis affecting natural teeth, the mean MPO concentration was 0.683 and 0.875 U/L, respectively, but in cases of dental implants with inflammation, the mean value was 0.622 U/L. Significant differences were reported between the two research groups when the total MPO levels, total nitrite levels, and total nitrite concentration were compared [53]. 

Sclerostin, TNF-related weak inducer of apoptosis (TWEAK), receptor activator of nuclear factor kappa-beta ligand (RANKL), and osteoprotegerin OPG levels in periodontal and peri-implant tissues in disease and health conditions were assessed by Yakar et al. in a cross-sectional study using GCF and PICF fluid. In comparison to the peri-implant health group, the peri-implantitis group displayed significantly higher levels of Sclerostin, TWEAK, RANKL, and OPG. The periodontitis group also had significantly higher levels of TWEAK, RANKL and OPG than the periodontal health group did. Moreover, statistically significant associations between several clinical parameters (probing depth, gingival recession, gingival bleeding time index, gingival index, and plaque index) and biochemical markers were discovered. However, further research is needed for an accurate interpretation of the dynamic changes in cytokine profile [54].

### 3.5. Inflammatory Markers in Saliva

IL-1β appears to play a key function in the inflammatory process among the several cytokines that are responsible for the initiation and modulation of host responses in inflammation. IL-1β has a significant role in inflammatory periodontal disease, according to research by Kornman et al. They found that an increase in salivary IL-1β predisposes a patient to chronic periodontitis by triggering an excessive inflammatory immune response [36,55].

The appearance of gum allergies and long-lasting black lines on the neck of restorations after fixed prostheses could be due to the mouth cavity saliva’s tendency to corrode metals. These side effects eventually result in a bad aesthetic aspect [40,56].

### 3.6. Inflammatory Markers in Blood

In Zhang’s study, hs-CRP and TNF-α blood levels showed a considerable reduction one day after fixed restorations were applied, in comparison with the pre-treatment values [40].

In patients with peri-implantitis, the blood levels of IL-6 and CRP were assessed. Mean CRP levels were found to be 0.795 mg/dL and 0.294 mg/dL in patients with peri-implantitis, respectively, in the control group. Mean IL-6 levels were found to be 12.178 pg/mL in the peri-implantitis group and 6.458 pg/mL in the control group, respectively. The statistical analysis revealed that the peri-implantitis group exhibited significantly higher levels of CRP and IL-6. The study concluded that elevated levels of CRP and IL-6 are associated with increased periodontal inflammation in peri-implantitis patients [57] (Table 4).

As highlighted by the scientific literature (Table 1, Table 2, Table 3 and Table 4), fixed dental prostheses could influence the expression of inflammatory markers and periodontal status. Nevertheless, as shown by this review, the results are highly heterogenous, making it impossible to arrive at any definitive findings at this time. Our review has demonstrated a diversity of perspectives on the impact of fixed dental prosthesis on inflammatory markers’ expression and periodontal health, sometimes providing contradictory results. This encourages additional research regarding this issue by carefully designed, comprehensive, longitudinal, or randomized clinical studies that might generate stronger conclusions.

## 4. Summary, Conclusions and Future Perspectives

Given the primary objective of our study, we may conclude that the presence of fixed dental prostheses influences the levels of inflammatory markers in gingival crevicular fluid, peri-implant crevicular fluid, blood and saliva, either positively or negatively. Regarding the periodontal status, there is evidence that some clinical parameters are modified in the presence of fixed prosthetic restorations. In the future, more comprehensive and more complex research is needed, with higher patient samples, since the research so far has led to either contradictory or incomplete results.

The various inflammatory markers found in periodontal disease patients and healthy people are promising biological indications that point to a viable method for predicting, diagnosing, and implementing personalized periodontal therapy. Collaboration between prosthodontists and periodontists is essential for a good treatment outcome since it will increase the restoration’s lifespan, enhance periodontal health, and improve the quality of life for dental patients.

## Figures and Tables

**Table 1:** Relevant studies regarding periodontal parameters and the presence of fixed dental prostheses.

Reference	Type of Study	Number of Patients	Main Findings
Ercoli et al. [5], 2018	Narrative Review	N.A.	The restoration’s design, fabrication, delivery and materials have often been associated with plaque retention and loss of attachment.
Silness et al. [14], 1982	Prospective follow-up study	16	Bacterial plaque is essential for the production of inflammation of the mucosa subjacent to bridge pontics.
Kancyper et al. [15], 2001	Prospective follow-up study	30	In patients with suitable oral hygiene, tooth-supported and implant-supported crowns with intracrevicular margins were not predisposed to unfavorable gingival responses
Kim et al. [16], 2021	Cross-sectional Survey	12.689	The number of prosthetic crowns is directly related to the prevalence of periodontitis.
Basnyat et al. [17], 2015	Prospective follow-up study	50	Single crown placement had no significant difference on plaque index and gingival index of the patient after 14 days and six months, whereas fixed partial denture showed significant difference.
Knoernschild et al. [18], 2000	Review	N.A.	Crowns and fixed partial dentures increased the incidence of advanced gingival inflammation adjacent to restorations, particularly if restorations had intracrevicular finish line placement, poor marginal adaptation, or rough surfaces. Both restorations in general did not accelerate the rate of adjacent bone loss.
Heidari et al. [19], 2019	Prospective follow-up study	23	Stainless steel crowns with proper fit have no adverse effect on gingiva of permanent first molars given that the patient maintains a good oral hygiene.
Yeasmin et al. [20], 2022	Prospective comparative experimental study	106	Periodontal health outcome of full veener crown in diabetic patients is adversely affected compared to that in non-diabetic patients.

N.A.—Non Applicable.

**Table 2:** Relevant studies regarding the presence of inflammatory markers in gingival crevicular fluid in patients wearing fixed dental prostheses.

Ref.	Type of Study	Number of Patients	Most Relevant Points
Abo-Elmagd et al. [2], 2021	Prospective observational study	24	At 2 weeks following the application of fixed dental prosthesis, IL-1β showed the lowest values.
Passariello et al. [6], 2012	Prospective follow-up study	74	IL-1β, but not IL-6 and TNF-α, showed enhanced levels at sites reconstructed with metalo-ceramic crowns.
Heboyan et al. [34], 2020	Prospective follow-up study	105	After the placement of dental crowns, gingival crevicular fluid’s pH increases in comparison with the initial value, both in the first six months and a year later following the cementation.
Koth et al. [35], 1982	Prospective follow-up study	54	Gingival inflammation surrounding full crown restorations may be controlled regardless of gingival margin placement when the gingiva is healthy, the restorations are adequate, and the patient is in a strict recall program.
Saravanakumar et al. [36], 2017	Prospective follow-up study	20	The zirconia crowns showed the lowest levels of IL-1b at the end of the three-month analysis.
Aggarwal et al. [37], 2022	Cross-sectional study	20	Preformed zirconia crown can be a relative replacement of stainless-steel crowns in primary molars as it revealed lower IL-1b values, less inflammation and with an advantage of aesthetics.
Chang et al. [38], 2014	Cross-sectional study	12	Differences in GCF IL-1α and IL-8 concentrations were observed when comparing fixed crown restorations with equigingival and supragingival margins.
Ariaans et al. [39], 2016	Cross-sectional study	49	The concentrations of IL-1b, IL-1ra and MMP-8 were not significantly different between restored teeth and controls or between lithium disilicate and zirconia restorations.
Zhang et al. [40], 2022	Prospective follow-up study	105	YKL-40, resistin, AST, and ALP levels in both groups increased one year after restoration compared with those before restoration, with lower levels in the zirconium crown group compared with the metal-ceramic crown group
Shang et al. [41], 2014	Prospective follow-up study	46	Fixed dental prostheses substantially raised the amount of GCF TNF-a, IL-6, sulcus bleeding index, and probing depth.
Mathew et al. [42], 2022	Prospective follow-up study	10	At the end of the fourth trial week, the TNF-α level was still significantly increased, probably due to gingival retraction.

**Table 3:** Relevant studies regarding the presence of inflammatory markers in peri-implant crevicular fluid in patients wearing fixed dental prostheses.

Ref.	Type of Study	Number of Patients	Main Findings
Wehner et al. [51], 2022	Pilot study	22	While CRP, TGF-β, IL-1β, IL-6, IL-8, MIP-1a, osteopontin, and osteoactivin protein expression all significantly decreased throughout the 6-months observation period, MMP-13 levels increased.
Yaghobee et al. [52], 2013	Cross-sectional study	32	There is a positive correlation between the IL-1bβ level and clinical parameters, such as plaque index, gingival index, pocket depth, and bone loss.
Kulkarni et al. [53], 2016	Cross-sectional study	42	Significant differences were reported between dental implants and healthy teeth research groups when the total myeloperoxidase levels, total nitrite levels, and total nitrite concentration were compared.

**Table 4:** Relevant studies regarding the presence of inflammatory markers in saliva and blood of patients wearing fixed dental prostheses.

Ref.	Type of Study	The Location where the Samples Were Collected	Number of Patients	Main Findings
Ditrichova et al. [56], 2007	Cross-sectional study	Saliva	25	Patients with oral lichenoid lesions underwent patch testing, and it was discovered that the majority of contact allergies were related to dental metals (cobalt, chrome, nickel, mercury, etc.).
Zhang et al. [40], 2022	Prospective follow-up study	Blood	105	Significantly lower values of hs-CRP and TNF-α were present in both groups after treatment compared with the levels before treatment, and compared with the metal-ceramic crown group, both inflammatory markers’ concentrations were lower in the zirconia crown group after treatment, with statistical significance.
Knichy et al. [57], 2021	Case-control study	Blood	40	Enhanced periodontal inflammation in peri-implantitis patients is accompanied by a considerable increase in the venous concentration of CRPs and IL-6.

## Data Availability

Data used to support the findings of this study are available from the corresponding author upon reasonable request.
